# Multiparametric MR mapping in clinical decision-making for diffuse liver disease

**DOI:** 10.1007/s00261-020-02684-3

**Published:** 2020-08-05

**Authors:** Helena B. Thomaides-Brears, Rita Lepe, Rajarshi Banerjee, Carlos Duncker

**Affiliations:** 1 Perspectum, Gemini One, 5520 John Smith Drive, Oxford, OX4 2LL UK; 2 Texas Liver Institute, 607 Camden St, Suite 101, San Antonio, TX 78215 USA; 3 Perspectum, 600 N. Pearl St. Suite 1960, Plaza of The Americas, Dallas, TX 75201 USA

**Keywords:** Multiparametric magnetic resonance imaging, cT1, Elastography, Diffusion-weighted imaging, T2*, PDFF

## Abstract

Accurate diagnosis, monitoring and treatment decisions in patients with chronic liver disease currently rely on biopsy as the diagnostic gold standard, and this has constrained early detection and management of diseases that are both varied and can be concurrent. Recent developments in multiparametric magnetic resonance imaging (mpMRI) suggest real potential to bridge the diagnostic gap between non-specific blood-based biomarkers and invasive and variable histological diagnosis. This has implications for the clinical care and treatment pathway in a number of chronic liver diseases, such as haemochromatosis, steatohepatitis and autoimmune or viral hepatitis. Here we review the relevant MRI techniques in clinical use and their limitations and describe recent potential applications in various liver diseases. We exemplify case studies that highlight how these techniques can improve clinical practice. These techniques could allow clinicians to increase their arsenals available to utilise on patients and direct appropriate treatments.

## Introduction

Chronic liver disease leads to progressive injury from various aetiologies such as: iron overload, steatosis, steatohepatitis, viral hepatitis, autoimmune, metabolic disease and some drug toxicities. These represent the majority of aetiologies of liver diseases and vary in estimated worldwide prevalence so that of 112 million prevalent cases of compensated cirrhosis reported in 2017, 36 million were due to hepatitis B, 23 million from alcoholic liver disease and 4 million from non-alcoholic steatohepatitis [1].

In recent clinical practice, the number of referrals for abdominal and liver magnetic resonance (MR), including focussed liver MRI for diffuse disease, has increased. In part this is because tools used in current clinical practice for the diagnosis of liver disease have intrinsic limitations. Screening, monitoring and therapy decision-making abilities of multi-parametric MRI (mpMRI) are being increasingly recognised by hepatologists and gastroenterologists. Liver biopsy on the other hand is invasive, associated with potential complications [2–4] and subject to variability in sampling and interpretation [5–7]. Serum biomarkers are widely available but are non-specific [8–11]. Transient elastography (TE) and controlled attenuation parameter (CAP) are available in many practices as point of care to assess elasticity of the liver and steatosis, respectively, but are limited by high measurement failure rates especially in obese patients [12–16]. New methods and standardisation of imaging protocols have allowed radiologists to quantify features of liver parenchyma and alter practice and decision-making, although this is not yet fully reflected in clinical guidelines.

Current clinical guidelines for the management of non-alcoholic fatty liver disease (NAFLD) do not recommend routine screening for steatosis, even in high-risk groups in primary care, diabetes, or obesity clinics [17]. Instead NAFLD guidelines emphasise stratification of patients into those at low and high risk of fibrosis, with diagnosis based on the use of specific serum biomarkers and elastography-based techniques [17,18]. Liver biopsy is recommended in patients at higher risk of steatohepatitis or advanced fibrosis, or cirrhosis, to confirm diagnosis or to clarify disease aetiology [17]. Nevertheless, guidelines for diabetes management recommend diagnostic procedures to assess the degree of NAFLD or non-alcoholic steatohepatitis (NASH) irrespective of liver enzyme levels, due to the high risk of liver disease progression in this patient population [19,20]. In autoimmune hepatitis (AIH), clinical guidelines recommend a combination of blood tests and biopsy to assess response to pharmacotherapy and monitor resolution of histological inflammation [21]. This can lead to disease mismanagement or complications for the patient that an imaging test has the potential to avoid. In genetic haemochromatosis, the guidelines centre around early detection and monitoring via blood tests and confirmation, if necessary, with biopsy [22].

The needs of payers, clinicians and healthcare systems that support patients through disease progression and prevention, however, go beyond the existing guidelines and highlight diagnostic gaps. The needs in the management of chronic liver disease are largely dictated by costs and availability of treatment options. Academic and real-world community clinical management varies due to availability of resources, with community clinicians less likely to follow guidelines or monitor patients frequently [23,24]. In outpatient clinics, differentiation of steatosis from steatohepatitis to identify severe disease in alcoholic liver disease, NAFLD or both (BAFLD) is extremely vital in the management of these patients [25]. In AIH and haemochromatosis early diagnosis and monitoring response to disease treatment is important. In addition, recent developments in our understanding of liver disease, in light of increased epidemics of obesity and autoimmunity, have increased the impact of chronic liver disease in related clinical care pathways, for metabolic (dysfunction) associated fatty liver disease (MAFLD) [26], for example. This places additional pressure on an already overloaded healthcare system but may be transformed by additional diagnostic tools, especially as the maturation of new pharmacotherapies in steatohepatitis and diabetes could introduce new treatment and diagnostic opportunities.

Recent developments suggest that mpMRI could bridge some of these diagnostic gaps, the biggest impact being to obviate the need for histological assessment of disease activity and staging via liver biopsy in many clinical disease states [22,27–36]. It is only a matter of time before liver mpMRI enables clinical practice transformation, following in the footsteps of success stories already seen in breast cancer, prostate cancer and cardiology [37–39]. Here we review some of the parametric MRI techniques in clinical use that underpin mpMRI, as well as some of their potential clinical limitations and describe recent potential applications in patients with chronic liver disease.

## mpMRI methods in clinical practice

Multiparametric MRI refers to use of multiple quantitative (parametric) MRI features or measures with several possibilities for combinations [34–36,40–45]. Therefore, these combinations could be used to evaluate two or more specific characteristics of chronic liver disease and diffuse liver processes, to include derivation of composite metrics [44,46]. We will limit this review to parametric MRI techniques in clinical decision-making for chronic liver disease that are available to the practicing radiologist. For example, T1rho is an exciting approach that explores a relaxation due to low frequency (kHz) exchange interactions and may find utility once it has been validated and deployed at scale [47], SWI (susceptibility weighted imaging) provides an approach for measuring local magnetic susceptibility that can assess fibrosis [41] and has been shown to be affected by iron, fat and collagen deposition [41,48,49]. Developments utilising contrast agents are not included, as these have been reviewed recently elsewhere [50] and are mainly used in characterisation of hepatocellular lesions and carcinomas.

- T2/T2*: Since R2 = 1/T2 and R2* = 1/T2*, we will talk about them indiscriminately. Iron content can be measured using spin-density projection-assisted R2 [51,52] or T2* transverse relaxation, for example with GRE sequences [16,28,40,53]. These methods are standardised across scanners [42,51] and commercially available (Resonance Health, Australia and Perspectum, UK, respectively). Semi-automated post-processing services with same day turnarounds are now possible for T2*. Fibrosis, fat, and other hepatic cellular pathology contribute to R2 and R2* and interfere with liver iron content estimation [54–56]. The effect of fat has accuracy implications in NAFLD [55] but appears to be relatively small and may be minimised by mathematical correction [57,58]. In, addition, R2* can be obtained simultaneously with PDFF with Dixon-based sequences [55,56,59]. The confounding effect of fibrosis may be overcome with newer processing methods [54].
- *Proton density fat fraction (PDFF)*: PDFF is a ratio, expressed as a percentage, of the fraction of the MRI-visible protons attributable to fat divided by all MRI-visible protons in that region of the liver attributable to fat and water. Taking advantage of the chemical shift between fat and water, pulse sequences can be used to acquire images at multiple echo times at which fat and water signals have different phases relative to each other [60, 61]. PDFF can be performed with very high precision using a multiple echo spoiled GRE sequence with > 3 echo times. To avoid biasing the PDFF measurement it is important to image with a low flip angle to minimise T1 weighting (such as flip angle 5°, TR = 12 ms at 1.5T,flip angle 3°, TR = 14 ms at 3T) [62]. A complete set of sequence recommendations has been formulated by the quantitative imaging biomarkers alliance group (QIBA) [63,64]. MRI-PDFF is easier to perform, has high reproducibility [65, 59, 64] and reflects fat distribution on at least one cross-sectional slice rather than a few voxels, so has replaced measurement of triglyceride content using ^1^H MR spectroscopy even in guideline recommendations [17, 66] including for diabetes [19]. To minimise T1 bias (fat has shorter T1 than water), a low flip angle is used, along with acquisition or algorithmic corrections for T2* effects [59,67–69]. Developments in echo times [70–72] and processing improve sensitivity to field inhomogeneities, signal-to-noise ratios and sensitivity at PDFF > 50% [73,74]. These also highlight that PDFF accuracy is not meaningfully confounded by any of age, sex, BMI, inflammation or fibrosis [75,76].
- *Magnetic resonance elastography (MRE)*: MRE uses low frequency mechanical shear waves to cause liver vibrations that are detected by MRI, based on a modified phase contrast pulse sequence [34, 77]. 3D-MRE takes advantage of additional spin-echo echo-planar-imaging (SE-EPI) to capture shear wave displacements along three dimensions, and images the entire liver rather than regions of interest (ROI) but to date is not FDA-cleared [78,79]. MRE is commercially available on 1.5T and 3T MRI scanners once suitable hardware is added in order to produce the requisite mechanical waves, and once specific software is installed for elastogram acquisition (Resoundant Inc., USA). Standardisation of 2D-MRE exists on three major vendors [80,81], although different shear wave frequencies are used outside the USA that are not FDA-cleared [82] and there is no consensus yet on the standards for ROI number, size or shape, which can add to measurement variability [83]. This could be overcome by dedicated freehand ROI selection under supervision of an experienced radiologist or by using additional software to aid in this process [84]. The accuracy of MRE for early fibrosis is reportedly superior to transient elastography (TE) but equivalent in cases of advanced fibrosis [34,85–87], and MRE shear waves may propagate through small- and medium-sized ascites. MRE has a lower measurement failure rate than TE and has reportedly better repeatability [88]. However, iron deposition in the liver is a reported confounder for MRE that is significantly associated with measurement failure [77]. MRE is confounded by even mild iron overload, necessitating mpMRI with T2, T2* or, more recently, SE-EPI sequences [35,89,90]. Additional measurement of PDFF to evaluate steatosis has been attempted alongside MRE [36,40], as PDFF and T2* can be acquired within a single breath-hold. As with TE, MRE values are affected by chronic and acute inflammation, which can cause overlap in elastography values of patients with no or mild fibrosis [9,91]. Thus, high liver stiffness values can be obtained without any degree of fibrosis, resulting in low positive predictive value.
- *T1/corrected T1 (cT1)*: Modified Look-Locker inversion recovery (MOLLI) T1 maps provide diagnostic information in the heart, so that increased T1 can be diagnostic of oedema (increased tissue water) or increased interstitial space [92–94], whilst increased extracellular volume is a powerful independent predictor of mortality in patients with severe aortic stenosis [95]. Similarly, the T1 of the water component is of diagnostic significance in the liver using MOLLI mapping or inversion recovery echo-planar imaging readouts to characterise tissue [28,68,96–98]. Since iron is a ferromagnetic material, it can shorten tissue T1 and T2 relaxation times and this is further accentuated by the dependence of MOLLI T1 on T2 [99], with potential bias equivalent to one fibrosis stage when hepatic iron content increases from normal to high levels (1.0 to 2.5 mg/g) [100,101]. The confounding effect of iron on T1 mapping is corrected by a compensatory algorithm, based on the application of a multi-compartment model to simulate tissue and water environments in the liver during changes in iron content and in extracellular fluid (a proxy for fibrosis) [100]. For simplification the resulting cT1 is treated as a parametric component in this review, despite requiring T2* measurement. cT1 is commercially available as post-processing software (with T2* and PDFF, Liver*MultiScan*™, Perspectum, UK) and correlates with parenchymal fibrosis, inflammation and ballooning [16,28,31,32,76,102]. cT1 shows low measurement failure rates, high repeatability and reproducibility that are superior to those of elastography techniques in both published and preliminary data [42,80,88]. Fat has some additive effect on MOLLI T1 measurements at 3T [103] and by extension on cT1, but optimisation of MOLLI sequence parameters may be used to manage these biases, for example by use of asymmetric echo times during bSSFP [104]. Correlation of cT1 with histological disease features is maintained even after controlling for steatosis [76].
- *Diffusion-weighted imaging (DWI)*: Quantitative measures of diffusion can be produced by measuring the magnitude (apparent diffusion coefficient; ADC) and directionality (fractional anisotropy) of diffusion. The accumulation of steatosis, inflammation and fibrosis can lead to changes in water diffusion and these can be measured using various DWI techniques. Whilst mainly applied clinically in focal lesion characterisation, recent developments have potential utility in viral hepatitis and staging fibrosis [105–110]. Limitations include lack of standardisation with inconsistencies reported for field strength [111] and B values [112,113]. Imaging homogeneity artefacts can be improved with simultaneous multi-slice respiratory-triggered acceleration (SMS-RT-DWI) [114]. Another DWI approach with promise is IVIM (intravoxel incoherent motion) that utilises diffusion imaging methods to explore both microcirculatory water motions and diffusion. Although specialist processing tools are required, recent studies indicate IVIM may further enhance fibrosis staging but this requires validation beyond focal disease [115–118].

Commercially available methodologies used in clinical practice differ in their performance and applicability to different MR scanner systems, with particular consequences in terms of operability (Table 1). Below we will describe how these techniques can be used for diagnosis, monitoring, and predicting outcomes—we will describe these in frequently occurring hepatology problems encountered in clinical care, with examples.

**Table 1:** Summary of characteristic features of commercially available MRI techniques used in clinical practice for chronic liver disease

Technology characteristic	R2 (FerriScan®)	cT1-T2*-PDFF (Liver*MultiScan*™)	MRE
Turnaround time	2-day service	1 h service	Point of care
Required hardware	1.5T MRI scanner	MRI scanner, multiple field strengths and manufacturers	MRI scanner and driver device to generate mechanical waves
Regulatory clearance	CE; TGA; FDA(510 k) and as Companion Diagnostic Device	FDA (510 k); CE; TGA; SMDR; NZ: listed on MEDSAFE	FDA (510 K) for 2-D MRE only
Standardisation of hardware (CoV)	Manufacturer-based biases reported for R2 and R2* [188]	For cT1 3.3% (Siemens, Philips) [88] For T2* 6.6% (Siemens, Philips) [88] For PDFF 0.8% (Siemens, Philips) [88]	10.7% (Philips, GE) [80]. 9.2–11.5% across field strengths (Siemens) [189]. Variability in mechanical waves used (40 Hz, 50 Hz, 60 Hz) [82]
Diagnosis of iron overload	High sensitivity (0.85–0.94) and specificity (0.92–1.00) for wide range of liver iron concentrations [52,190]	T2* AUROC for stainable iron: 0.79–0.94 [16,28]	No, a confounder that can be overcome by T2* or SE-EPI sequences [35,89,90]
Diagnosis of NASH and disease activity	Not applicable	cT1 AUROC for NASH: 0.69–0.72 [85,102] cT1 AUROC for NAFLD: 0.93 [102] cT1 AUROC for ballooning: 0.84 [adapted from [32]] cT1 AUROC for NAS ≥ 5: 0.74 [102]	AUROC for NASH: 0.58 [85]
Diagnosis of steatosis	Volumetric fat fraction of liver tissue (% fat) rather than PDFF available as HepaFatScan	PDFF AUROC for steatosis grades: ≥ G1: 0.93 [85] ≥ G2: 0.96 [85] ≥ G3: 0.94 [85]	Not applicable but can add PDFF [36, 40]
Diagnosis of fibrosis	Not applicable but can combine with T1 or DWI or MRE [35,41,45,89]	cT1 AUROC for fibrosis stages: ≥ F2: 0.63–0.79 [32, 85, 102] ≥ F3: 0.62–0.74 [32, 85, 102] F4: 0.72–0.85 [16] [adapted from [32]]	AUROC for fibrosis stages: ≥ F2: 0.83 2D-MRE [85] ≥ F3: 0.96 2D-MRE [85] F4: 0.91 [82]
Diagnosis of high risk	No	AUROC for NASH or fibrosis ≥ F2: 0.83 [102]	
Coverage of liver	Whole slice analysis over 11 slices based on ROI	Whole slice analysis over 1–4 slices based on ROI or segmentation	Whole slice analysis over 4 slices based on ROI
Measurement failure rate	Not reported	1.4–5.6% [16, 32, 102]	4.3% [77, 82]
Repeatability (CoV)	15–21% [51]	For cT1 1.7–3.3% [16, 42, 88] For T2*2.6- 5.5% [16, 42] For PDFF 0.8–8.8% [16, 42]	11.0% [88]
Confounded by iron	Not applicable	No, cT1 corrected for iron [28, 100]	Yes, requires separate quantification of iron [77, 89]
Confounded by fat	Yes, PDFF can influence R2* at both 1.5T and 3T [55, 57]	Yes, for cT1 but can be managed by altering sequence parameters [103, 104]	Yes, in paediatric populations [161]
Confounded by comorbidities	Yes, fibrosis [54]	Yes, inflammation and fibrosis both increase cT1 [16]	Yes, by inflammation, passive congestion and large ascites [9, 77]

## Applications of mpMRI in chronic liver disease

### Haemochromatosis

The proper staging of patients with genetic heamochromatosis (HFE) is paramount for treatment. The ability to use MRI to quantify liver iron concentration and the presence of non-invasive serologic markers for fibrosis prediction (serum ferritin, platelets, transaminases), have diminished the diagnostic need for biopsy in haemochromatosis. Genetic testing is required to differentiate true genetic hemochromatosis (homozygous C282Y) from the other forms of milder hemochromatosis or even secondary iron overload syndromes [22,119]. Recent studies have identified elevated iron without homozygosity for the p.C282Y variant in the HFE gene, highlighting the continued undetected disease existing in the general population [120–122]. Increased iron can be co-existing in NAFLD, ALD and other chronic liver diseases [22,122,123]. Elevated liver iron has been reported in NALFD cohorts at prevalence that ranges between 10–34.5%, based on histochemical staining [40,102,124–126]. Both R2 and T2* based imaging could therefore be used clinically if integrated into clinical guidelines to identify such cases. Additional clinical applications for R2 and T2* are reviewed extensively by Wood [127].

Foci of increased iron occur in the regenerating nodules surrounded by fibrosis in cirrhotic livers [128]. The development of fibrosis and liver cirrhosis changes both the prognosis and the management of haemochromatosis, especially as patients improve after treatment with phlebotomy and show liver fibrosis regression [22]. Detection of fibrosis through cT1 or TE is preferable to MRE in these cases due to the confounding effect of iron resulting in MRE technical failures [77].

### Steatohepatitis (NASH/ASH)

Alcohol acts synergistically with obesity and diabetes in the progression to cirrhosis and hepatocellular carcinoma [93], patients with both NAFLD and ALD have more advanced fibrosis compared to those with NAFLD alone [25]. Indeed, steatohepatitis from both NAFLD and ALD is associated with higher risk of cardiovascular disease and outcomes than either NASH or ASH alone [129, 130]. Hepatic fibrosis has been shown to predict patient mortality [131], so clinicians have relied on elastographic techniques to evaluate severity and make appropriate decisions.[78]. However, other histological features contribute to steatohepatitis, such as inflammation and ballooning, collectively known as disease activity [17]; steatohepatitis is defined as the presence of 5% steatosis with inflammation and hepatocyte injury (e.g. ballooning), with or without any degree of fibrosis. Most of these tissue characteristics may be detected as an increase in T1 relaxation time [31,96,98].

#### Diagnosis

The diagnostic accuracy, linearity and precision of PDFF has been validated in many studies, showing that PDFF assessments closely correlate with steatosis assessment based on liver biopsy, magnetic resonance spectroscopy and chemical analysis of tissue samples [30,64,132]. However, PDFF cannot differentiate simple steatosis (NAFL) from steatohepatitis (NASH) necessitating other measurements to identify disease activity and fibrosis. cT1 has been used to improve stratification of non-alcoholic fatty liver disease (NAFLD) in the general population [133], in patients with co-prevalent type 2 diabetes [134] and from other parenchymal disease [102], whilst MRE can also discriminate healthy from NAFLD individuals [135]. Thus, radiology could enable earlier detection of disease before increased severity or fibrosis develop.

The diagnostic accuracy of fibrosis using cT1 is either equivalent or inferior to diagnosis by elastography based on comparative studies in NAFLD-only cohorts, with equivalent AUROC of 0.83 reported for cT1-based diagnosis of NASH or significant fibrosis in predominantly NAFLD cohorts as published and preliminary data indicate [32,85,102]. This may reflect the relatively small percent change in collagen percentage area that differentiates between stages of fibrosis [7], especially as mouse models of liver fibrosis have demonstrated that T1 and T2^∗^ correlations to fibrotic disease severity vary with aetiology [136]. Accuracy improves when cT1 is applied as a composite biomarker with blood biomarkers (AUROC of 0.84–0.97 are reported for detection of NASH with significant fibrosis, as published and preliminary data indicate [46,137]).

Using MRE, the accuracy of diagnosis of fibrosis (AUROC of 0.84–0.93 reported, depending on the fibrosis stage) is better than for identification of NASH only (AUROC of 0.58–0.73), although higher accuracy has been reported in mainly fibrotic NASH cohorts [34,82,85,87,135,138,139]. 3D-MRE was shown to be superior to 2D-MRE for advanced fibrosis and equivalent for NASH [78]. MRE is recommended for identifying patients who are at risk for steatohepatitis and/or advanced fibrosis in guidelines.

Conversely, cT1 is superior to MRE in stratification of NASH as shown in NAFLD cohorts in the UK, US and Japan, as preliminary data show [140,141]. In NAFLD cohorts cT1 strongly correlated with NAFLD activity score [102] and ballooning [32], with an AUROC of 0.84 for diagnosis so it may have utility in earlier detection and diagnosis of disease activity. Limited correlation to lobular inflammation has been observed in NALFD cohorts [16,32,76,102], although moderate to severe inflammation significantly increased T1 independently from fibrosis, using cT1 (or echoplanar imaging [96] in cohorts of chronic liver disease of mixed aetiologies [28]. This may reflect the low grade inflammation seen in NAFLD [142]. Use of cT1 to confirm disease in patients with suspected NAFLD may be financially advantageous as a result of reducing the need for further confirmatory diagnostics such as liver biopsy in UK and European clinical care published and preliminary data show [29,102,134]. Fat droplets in NASH cause changes to the liver parenchyma including oxidative stress, activation of cytokines resulting in local inflammation and eventual collagen deposition [143]. Such effects could also affect the amount of extracellular water and free water diffusion and have been investigated with DWI [110,115]. Emerging research on the derivation of ADC and IVIM signals suggests that microcirculation in vessels (perfusion) rather than molecular diffusion within liver tissue may correlate with histological staging of liver fibrosis [115]. Standardisation of the acquisition protocols, based on respiratory-triggered fat-saturated spin-echo echo-planar imaging sequences, would serve to validate these findings, with potential application in diagnosis of inflammation.

#### Monitoring

**Fig. 1:**
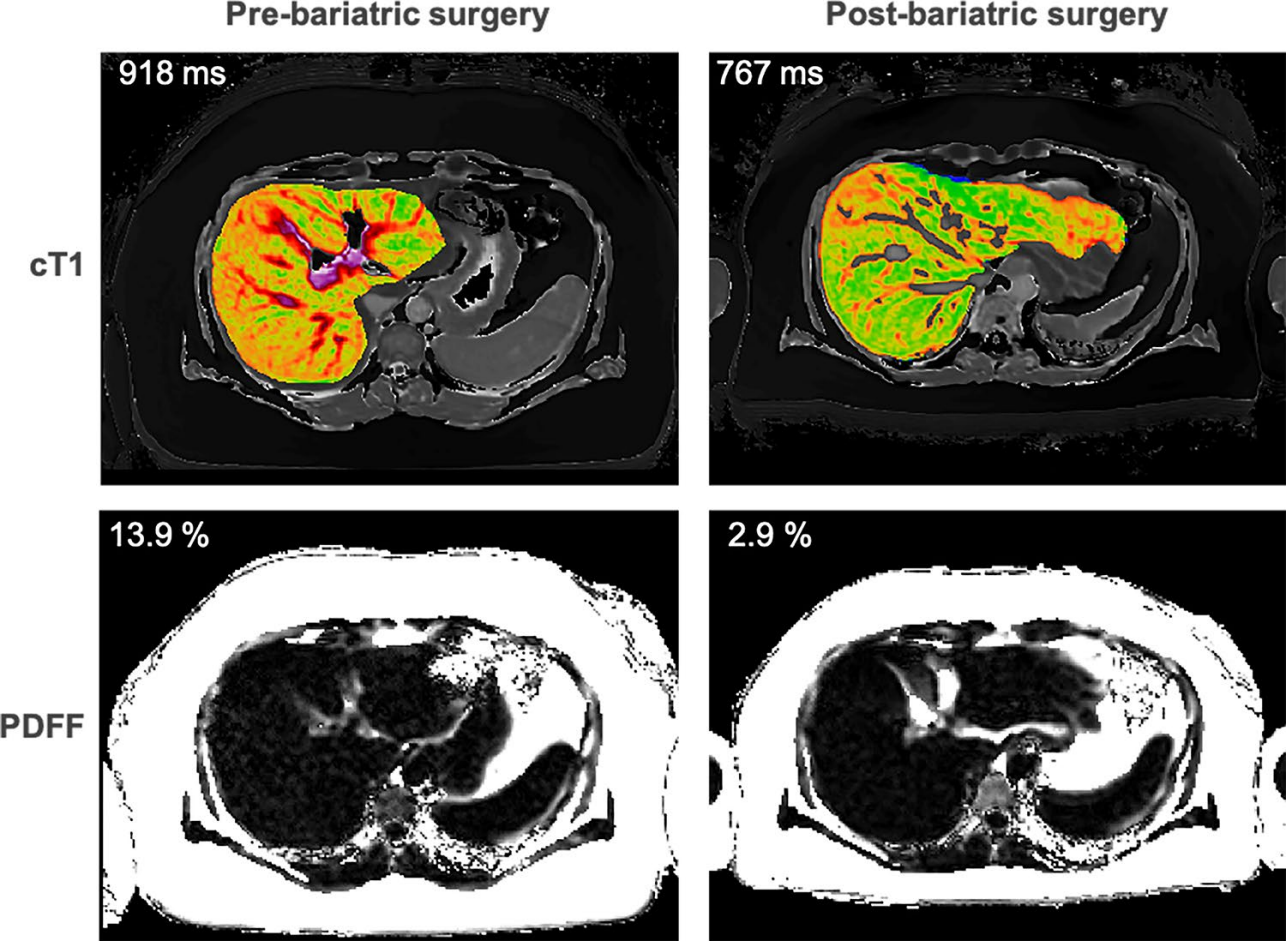
Example mpMRI case showing reduction in cT1 and PDFF values following bariatric surgery

Until now there have been no licensed pharmaceutical therapies for the NAFLD spectrum and, despite the difficulty of patient adherence to lifestyle changes, diet and exercise have been the first-line recommendations. Bariatric surgery is available for morbidly obese patients with steatohepatitis and can be monitored by PDFF, MRE or cT1 [36] (Fig. 1). However, not all patients are candidates for surgery. Clinical practice will likely change due to the encouraging interim results of a phase 3 study with obeticholic acid in reducing fibrosis [144], and other phase 3 trials affecting hepatic metabolism, or acting as anti-inflammatory or anti-fibrotics [145]. MRI is critical in clinical trials for NASH, with PDFF and cT1 being used as primary endpoints and MRE as a secondary endpoint in both published and preliminary findings [146,147]. A recent study of a chemical inhibitor of de novo lipogenesis, GS-0976, improved outcomes in NASH patients and reduced PDFF but no substantial change was detected using MRE [148].

#### Diagnosis of complications and predicting outcomes

Both PDFF and cT1 are significantly higher in patients with type 2 diabetes [149,150] and a positive correlation between MOLLI T1 values in the liver and history of cardiovascular disease has been reported in a large multi-ethnic, adult population study spanning over 10 years [151]. In a smaller study MRE confirmed the association between fibrosis and increased cardiovascular risk in patients with type 2 diabetes [152]. cT1 can be used to predict liver-related clinical outcomes (such as ascites, encephalopathy, liver-related mortality and hepatocellular carcinoma) with 100% negative predictive value [31] and as accurately as biopsy [153] in patients with ASH, NASH or viral hepatitis. cT1 can also be used to predict liver event-free survival as emerging data indicate [154,155]. cT1 has reported application in detecting portal hypertension as published and preliminary data indicate [156–158] and MRE in predicting variceal bleeding in cirrhotic patients [159, 160]. Both technologies could provide additional value in prediction of liver-related outcomes of patients after hepatic resection or transplantation.

#### Paediatric disease

**Fig. 2:**
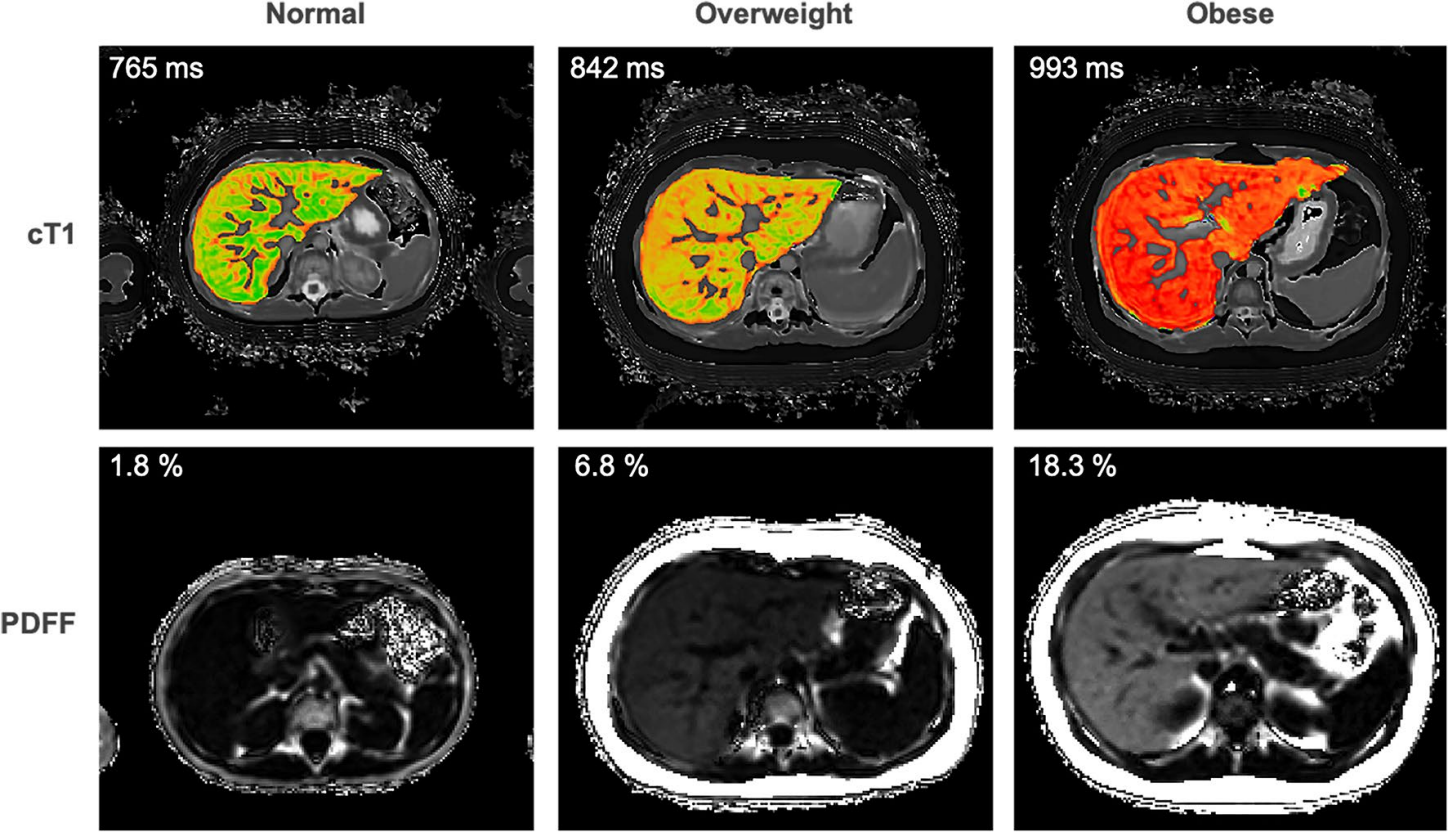
Example mpMRI cases of cT1 and PDFF in normal, overweight and obese paediatric cases

Histopathological features of NAFLD in children may differ from those in adults, particularly in younger children in whom steatosis may be more abundant or accentuated in different zones. Inflammation and fibrosis may be concentrated in portal tracts initially rather than the traditional pericentral seen in adults and ballooning is less frequent as well. The presence of significant steatosis or inflammation in a biopsy-confirmed fibrotic cohort spanning infants to young adults resulted in a significant reduction in MRE sensitivity [161]. AUROC for significant fibrosis dropped from 0.82 to 0.53 in the presence of steatosis [161], whilst in contrast data with cT1 confirms that higher disease activity is present with increasing obesity in emerging data [162] (Fig. 2). As with adults cT1 in paediatrics has high repeatability and reproducibility [163] and correlates with histological scoring of ballooning, fibrosis, and both portal and lobular inflammation [164], as suggested by preliminary data.

### Viral hepatitis

**Fig. 3:**
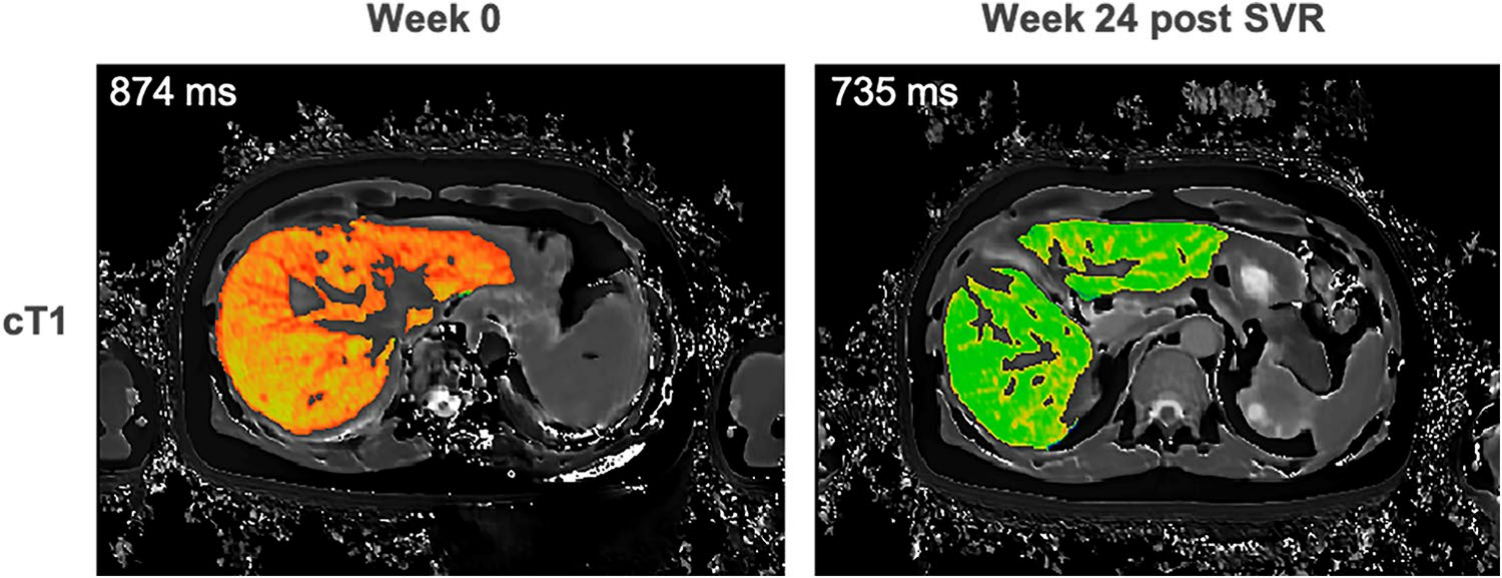
Example mpMRI case showing reduction in cT1 following sustained viral response (SVR) to 24-weeks of antiviral treatment for Hepatitis C

Clinicians can stage fibrosis and level of inflammation with MRE or cT1 [28,31,102] and monitor effect of treatment [165], which is important to know prior to therapy decision-making for Hep C and Hep B. (Fig. 3). Compared to steatohepatitis, viral disease results in higher incidence of cirrhosis. Elevated T1 in the liver has been associated with increasing severity of cirrhosis using modified respiratory-triggered inversion-recovery sequences [98]. High T1 values could stratify compensated cirrhosis from decompensated cirrhosis and were associated with and predictive of liver disease outcomes in patients with compensated cirrhosis [98]. In addition, DWI, in particular IVIM, could stratify patients with viral hepatitis in terms of fibrosis [109,115,166] and inflammation [105,110,116], with recent publications discriminating cirrhotic livers from healthy livers [107,116] and stratifying disease severity based on blood biomarker scoring systems [106] or TE [167]. DWI also attains high accuracy for identification of oesophageal and gastric fundic varices [107]. However, as TE is more scalable in the developing world this is a more frequent diagnostic solution.

### Autoimmune liver disease (AIH, cholangitis)

AIH is a disease associated with the production of autoantibodies (ANA, SMA), resulting in chronic inflammation and, if untreated, fibrosis and cirrhosis over time [168]. AIH patients tend to experience disease flares throughout their life span. Patients require life-long monitoring to evaluate response to treatment options, such as corticosteroids and azathioprine among others [21]. Biochemical remission is often challenging, and only 38–93% of patients are able to achieve a complete histological response [169]. Up to 50% of patients develop cirrhosis while undergoing treatment despite [170] having normal biochemical markers [171].

#### Diagnosis

cT1 is more sensitive to subtle changes in inflammation in the liver than circulating biomarkers and elastography, as published and preliminary data show [172–175]. This supports the ability of cT1 to measure liver inflammation without steatosis [172–175]. High AUROC for detection of advanced fibrosis have been reported in patients with AIH, using MRE [176] or DWI [109]. Additionally, as primary biliary cholangitis (PBC) and primary sclerosing cholangitis (PSC) can be co-prevalent with AIH, cT1 can be used to differentiate between patients with AIH and biliary disease as emerging data show [177]. Furthermore, these patients may benefit from additional characterisation with magnetic resonance cholangiopancreatography (MRCP) [178] or DWI [167], including with recently developed software to enhance and quantitate MRCP images (MRCP+, Perspectum, UK) [179,180]. Additionally, cT1, MRE and DWI have reported application in detecting portal hypertension in such cohorts, with highest AUROC reported for MRE [157].

#### Monitoring

**Fig. 4:**
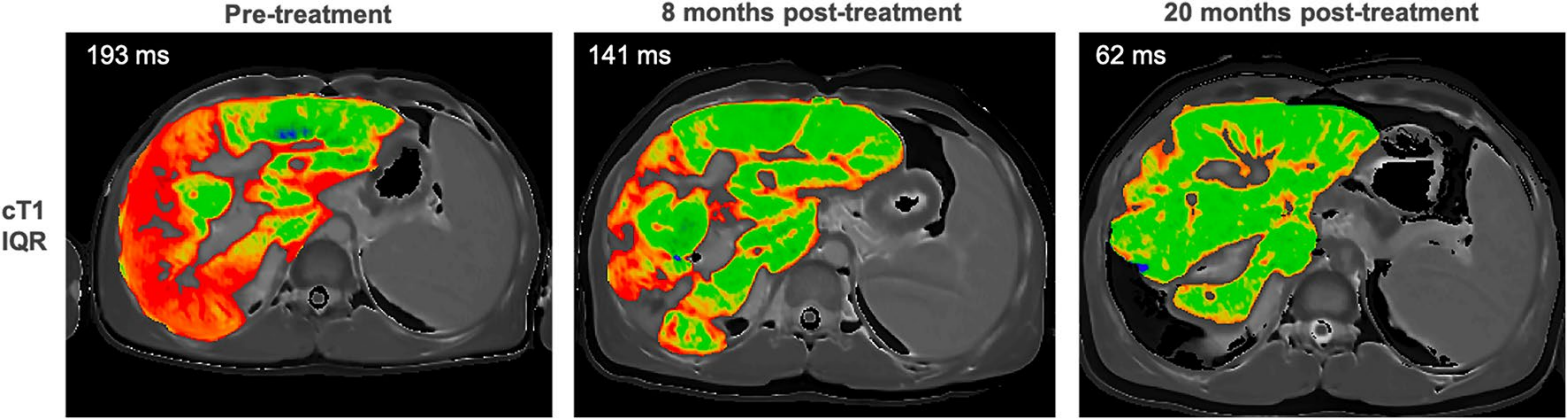
Example mpMRI case showing improvement in cT1 IQR following treatment in patient with concurrent AIH and primary sclerosing cholangitis

Improvements on immunosuppressive treatment (elevated baseline returning to normal levels at follow-up) have been detected by cT1 in AIH, and cT1 discriminates between treatment-naive AIH patients and those post-treatment, as published and emerging data show [173,181,182]. cT1 can be used to predict clinical outcomes (AUROC for future flare events 0.721, *p* = 0.003) better than TE (AUROC: 0.502, *p* = 0.983) and the enhanced liver fibrosis test (AUROC: 0.501, *p* = 0.992), indicated by emerging data [174,182,183]. Subtle changes in disease heterogeneity can also be quantified by cT1, as the interquartile range (IQR) (Fig. 4).

#### Paediatric disease

**Fig. 5:**
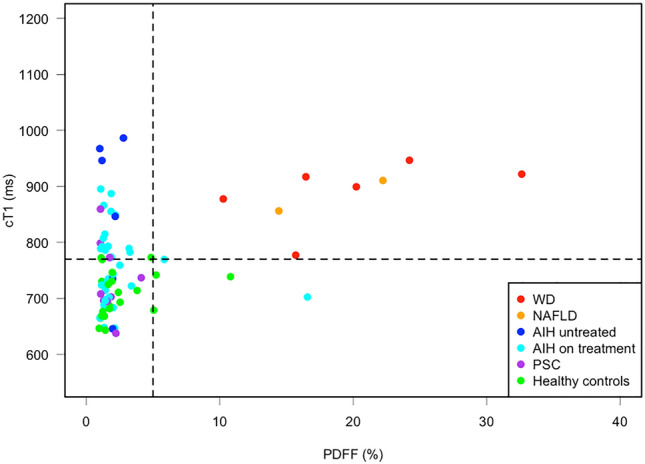
Scatterplot showing the distribution of cT1 and PDFF across paediatric disease groups and healthy controls, showing AIH patients on treatment, treatment naive AIH, Wilson’s disease (WD), primary sclerosing cholangitis (PSC) and NAFLD

In paediatric populations cT1 has shown significant correlations with ballooning, fibrosis and inflammation in emerging data [164,182,184]. When combined with circulating biomarkers, cT1 can predict flares in paediatric AIH with a specificity of 100% and sensitivity of 50% (PPV 100%, NPV 57%) [183]. Moreover, cT1 can be used to stratify patients with AIH from those with other liver diseases including Wilson’s disease in emerging data [183,185–187] (Fig. 5), thus suggesting a wide range of potential utilities. This stratification is further improved when cT1 is used as a composite biomarker with PDFF in these studies [181].

## Conclusion

The studies described here demonstrate that high accuracy can be obtained by application of an array of MRI techniques in clinical care. MRE supports stratification of fibrosis as a window to disease state, cT1 is diagnostic of disease activity and progression whilst iron content can be quantified by (reciprocal) transverse relaxation. These techniques have the potential to influence American College of Radiology guidelines and to complement existing diagnostics, enabling clinicians to diagnose, stratify and monitor liver disease earlier and with greater confidence. In particular, improved monitoring of response to new and existing treatments may also be possible for a variety of liver diseases. This may reduce the reliance on invasive liver biopsy and improve patient experience and outcomes.
